# Replication stress induces specific enrichment of RECQ1 at common fragile sites FRA3B and FRA16D

**DOI:** 10.1186/1476-4598-12-29

**Published:** 2013-04-22

**Authors:** Xing Lu, Swetha Parvathaneni, Toshifumi Hara, Ashish Lal, Sudha Sharma

**Affiliations:** 1 Department of Biochemistry and Molecular Biology, College of Medicine, Howard University, 520 W Street, NW, Washington, DC 20059, USA. 2Genetics Branch, National Cancer Institute, National Institutes of Health, Bethesda, MD 20892, USA; 2 Genetics Branch, National Cancer Institute, National Institutes of Health, Bethesda, MD, 20892, USA

**Keywords:** RecQ, Helicase, Replication stress, DNA repair, DNA damage, Genomic instability

## Abstract

**Background:**

Stalled replication forks at common fragile sites are a major cause of genomic instability. RecQ helicases, a highly conserved family of DNA-unwinding enzymes, are believed to ease ‘roadblocks’ that pose challenge to replication fork progression. Among the five known RecQ homologs in humans, functions of RECQ1, the most abundant of all, are poorly understood. We previously determined that RECQ1 helicase preferentially binds and unwinds substrates that mimic DNA replication/repair intermediates, and interacts with proteins involved in DNA replication restart mechanisms.

**Method:**

We have utilized chromatin immunoprecipitation followed by quantitative real-time PCR to investigate chromatin interactions of RECQ1 at defined genetic loci in the presence or absence of replication stress. We have also tested the sensitivity of RECQ1-depleted cells to aphidicolin induced replication stress.

**Results:**

RECQ1 binds to the origins of replication in unperturbed cells. We now show that conditions of replication stress induce increased accumulation of RECQ1 at the lamin B2 origin in HeLa cells. Consistent with a role in promoting fork recovery or repair, RECQ1 is specifically enriched at two major fragile sites FRA3B and FRA16D where replication forks have stalled following aphidicolin treatment. RECQ1-depletion results in attenuated checkpoint activation in response to replication stress, increased sensitivity to aphidicolin and chromosomal instability.

**Conclusions:**

Given a recent biochemical observation that RECQ1 catalyzes strand exchange on stalled replication fork structures in vitro, our results indicate that RECQ1 facilitates repair of stalled or collapsed replication forks and preserves genome integrity. Our findings provide the first evidence of a crucial role for RECQ1 at naturally occurring fork stalling sites and implicate RECQ1 in mechanisms underlying common fragile site instability in cancer.

## Background

Inherited syndromes characterized by the replication defects and genomic instability emphasize that DNA replication and repair must be highly coordinated processes [1]. Deficiencies in DNA replication and repair mechanisms become particularly detrimental at genomic sequences that present unique challenges to the replication fork progression [2,3]. Common fragile sites (CFS) are specific chromosomal regions, extending over large DNA sequences, which are especially prone to genomic instability under conditions of replication stress [4,5]. At fragile sites, under conditions of replication stress, the replicative polymerases may uncouple from the helicase, resulting in long regions of single-stranded DNA ahead of polymerase that could pose fork barriers leading to fork disassembly and DNA breaks [5,6]. CFS are expressed as site-specific gaps or breaks on metaphase chromosomes after partial inhibition of DNA synthesis, and exhibit sister chromatid exchange (SCE), deletions and translocations [5]. Thus, CFS serve as genomic hot spots for DNA damage and carcinogenesis [7].

Members of RecQ family DNA helicases, represented by five homologs (BLM, WRN, RecQ4, RecQ5β and RECQ1) in humans, are implicated in genome maintenance through repair of stalled or collapsed replication forks [8,9]. RecQ helicases are specifically recruited to the sites of arrested DNA replication where in addition to serving repair functions, they help stabilize stalled replication complexes and likely contribute to the S-phase checkpoints [10-14]. Importantly, WRN, defective in the premature aging disease Werner syndrome, has been shown to regulate fragile site stability [15]. WRN helicase activity and ATR-mediated checkpoint response collaborate in a common pathway to maintain CFS stability and WRN-deficiency is associated with accumulation of gaps and breaks at CFS [13,15]. In vitro, WRN enhances human DNA polymerase δ-dependent DNA synthesis through secondary structure forming sequences within the FRA16D fragile site [16]. Functions of BLM, defective in the cancer predisposition disease Bloom syndrome, are especially important for cells in dealing with replication stress [12]. WRN and BLM likely perform distinct functions in recovery from fork stalling genome-wide [17], and collective literature points to their significant roles in maintaining genomic stability at CFS. Loss of BLM induces 53BP1 nuclear bodies that guard chromosomal fragile sites against genomic instability [18]. Furthermore, BLM associates with the ultrafine DNA bridges that interlink sister chromatids at CFS loci and is required for their resolution [19]. In fact, human RecQ homologs exhibit diverse catalytic activities relevant to replication restart pathways including disruption of Rad51 presynaptic filament (BLM and RecQ5β) [20,21], dissolution of double Holliday junctions (BLM) [22,23], branch migration of Holliday junctions (BLM, WRN and RECQ1) [24-26] dissociation of mobile D-loops (BLM, RECQ1 and WRN) [23,27,28], and annealing of complementary single strand DNA (all five) [29-33]. Functions of RecQ helicases are therefore likely to be critical under replication stress; among these, the roles of WRN and BLM remain best characterized while there is only limited information available for other RecQ proteins.

RECQ1 (also known as RECQL or RECQL1), the most abundant of the five human RecQ proteins, was recently identified as an integral component of replication complex in unperturbed cells and implicated in maintaining replication fork progression after initiation [34]. Indeed, recombinant RECQ1 binds and unwinds model replication forks and DNA structure intermediates of recombination [33]. Most recently, RECQ1, similar to WRN, was also shown to promote strand exchange on synthetic stalled replication fork-mimicking structures in vitro [35]. Depletion of RECQ1 reduces cell proliferation and RECQ1-deficient cells accumulate DNA damage, display heightened sensitivity to DNA damaging agents that induce stalled and collapsed replication forks, and exhibit chromosomal instability [36-38]. Evidently, RECQ1-deficient cells are characterized by spontaneous Rad51 foci and elevated SCEs [38] reminiscent of recombinogenic structures proposed to arise upon replication fork restart following collapse [39]. To investigate a role of RECQ1 in replication fork maintenance, we employed quantitative chromatin immunoprecipitation (ChIP) and examined in vivo chromatin interactions of RECQ1 in cycling cells following replication inhibition with hydroxyurea or aphidicolin. We report specific binding of RECQ1 to two major fragile sites, FRA3B and FRA16D, in aphidicolin treated HeLa cells. Our results provide novel evidence and complement existing biochemical data for a role of RECQ1 in genome maintenance upon replication stress.

## Results

The cellular phenotypes of RECQ1-deficiency and biochemical data suggest a role of RECQ1 in mechanisms involved in replication stress response. To investigate a role of RECQ1 in the repair of stalled or collapsed replication forks, we have evaluated chromatin interaction of RECQ1 at specific genomic loci in cells exposed to replication blocking agents.

### Recruitment of RECQ1 to lamin B2 origin is selectively enhanced upon replication stress

**Figure 1:**
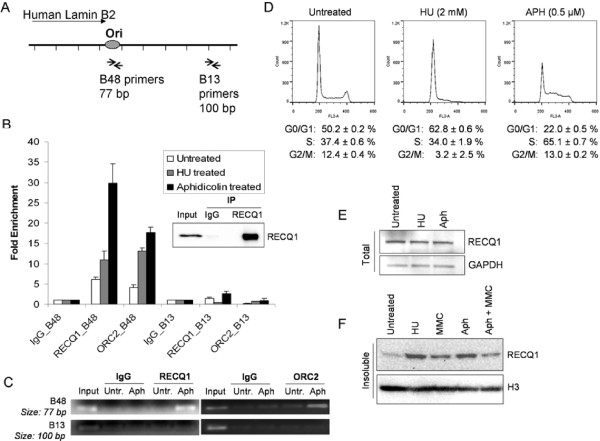
**Enhanced enrichment of RECQ1 at lamin B2 origin after replication stress. A**. Genomic regions containing the human lamin B2 origin of replication. Primer sets used for qPCR analyses of origin (B48) and non-origin (B13) containing DNA are indicated. **B**. Quantification of cross-linked lamin B2 origin immunoprecipitated using the indicated antibodies. HeLa cells were either untreated or incubated with APH (0.5 μM) or HU (2 mM) for 24 h and processed for ChIP using a RECQ1-specific antibody. ORC2 antibody was used as positive control for origin-enrichment, and rabbit IgG served as negative control. qPCR was performed with origin-specific and non-origin primers for the lamin B2 locus. Fold enrichment was determined over IgG and is shown for each primer pair. Results are expressed as means ± SEM for at least three independent experiments. Specificity of RECQ1 antibody in immunoprecipitation (IP) of endogenous RECQ1 from HeLa extract is shown; input represents 10% of the extract used for IP. **C***.* A representative gel of the amplified DNA immunoprecipitated with indicated antibodies. Input represents 1% of the cross-linked chromatin used for ChIP. **D***.* Cell cycle profiles of untreated and HU or APH treated (24 h) HeLa cells. Propidium iodide stained cells were analyzed by FACS for DNA content; distribution in the G_1_, S, and G_2_/M phases of the cell cycle is indicated. **E***.* Total RECQ1 protein level in HeLa cells following 24 h treatment with HU (2 mM) or APH (0.5 μM). GAPDH is loading control. **F***.* Replication stress-dependent chromatin association of RECQ1. Western blot detection of RECQ1 in chromatin-enriched fractions of untreated and APH (0.5 μM), HU (2 mM) or MMC (0.5 μg/ml) treated cells, or following pretreatment with APH for 4 h before incubation with MMC (16 h). Histone H3 is loading control and chromatin marker. APH, aphidicolin; HU, hydroxyurea; MMC, mitomycin C.

Silencing of RECQ1 by siRNA leads to reduced cell proliferation [37,40,41]. A key step in the regulation of cell proliferation is initiation of replication. DNA replication initiates from a defined initiation site, from which replication progresses in both directions. One of the best characterized metazoan origins is located at the 3’ end of the human lamin B2 gene (Figure 1A) [42]. RECQ1 binds to the lamin B2 origin in unperturbed cells and knockdown of RECQ1 results in reduced origin firing and defective replication elongation [34]. To test whether RECQ1 plays a role in mammalian fork progression after initiation, we investigated the effects of fork stalling on binding of RECQ1 to lamin B2 origin in HeLa cells by ChIP (Figure 1B, C). ChIP experiments were done using cells that were either untreated or treated with hydroxyurea which specifically inhibits class I ribonucleotide reductase depleting dNTP pools [43,44] or aphidicolin which is an inhibitor of DNA polymerase α, δ and ϵ [45,46] to induce replication forks arrest. Thus, replication stress induced by hydroxyurea (2 mM, 24 h) and aphidicolin (0.5 μM, 24 h) treatment is mechanistically different. Cross-linked chromatin was immunoprecipitated with a control IgG or a specific antibody against RECQ1 that has been successfully used to identify protein and chromatin interactions of RECQ1 (Figure 1B) [34,47]. Immunoprecipitation of ORC2, a known lamin B2 origin binding protein and a part of the pre-replication complex [42], served as positive control in these experiments. Following cross-link reversal, the immunoprecipitated chromatin was used to determine the lamin B2 origin-containing DNA as well as an adjacent region, B13 that does not contain origin by quantitative real time PCR (qPCR). ORC2 interacted with lamin B2 origin, and approximately 4-, 13- and 18-fold enrichment of lamin B2 origin-specific DNA was found in ORC2 immunoprecipitate as compared to IgG in untreated, hydroxyurea, or aphidicolin treated HeLa cells, respectively (Figure 1B). Lamin B2 origin-specific DNA was enriched nearly 6-fold in RECQ1-immunoprecipitate as compared to IgG in untreated HeLa cells (Figure 1B). Treatment with replication inhibitors induced a further enrichment of RECQ1 at lamin B2 origin; approximately 11- and 30-fold enrichment of lamin B2 origin specific DNA was obtained in RECQ1 immunoprecipitate as compared to IgG in cells treated with hydroxyurea or aphidicolin, respectively (Figure 1B). In comparison, minimal binding to the control B13 containing DNA was observed for ORC2 or RECQ1-immunoprecipitates (Figure 1B, C). Thus, RECQ1 binds to the lamin B2 origin of replication in unperturbed HeLa cells and replication stress significantly enhances origin binding of RECQ1.

RECQ1 protein is expressed throughout the cell cycle and is not regulated in cell cycle stage specific manner [48]; although a small increase is reported in T98G cells synchronized in S-phase using serum starvation or mimosine treatment [34]. We monitored cell cycle progression in HeLa cells utilized in ChIP assay by FACS analysis (Figure 1D) and also determined total cellular RECQ1 protein level (Figure 1E). Total RECQ1 protein level was not modulated appreciably in HeLa cells following treatment with hydroxyurea or aphidicolin (Figure 1E). We next examined cellular RECQ1 in chromatin containing fractions following treatment with agents that introduce replication fork blocking lesions. HeLa cells either untreated or treated with hydroxyurea (2 mM, 24 h) or aphidicolin (0.5 μM, 24 h) were subjected to detergent extraction to isolate insoluble nuclear pellet containing proteins that were tightly bound to chromatin and/or nuclear matrix. RECQ1 protein in untreated cells predominantly fractionated with soluble proteins and only a minor fraction associated with chromatin in the insoluble nuclear pellet (Figure 1F). Interruption of DNA synthesis by hydroxyurea or aphidicolin resulted in increased RECQ1 in the insoluble fraction that also contained histones (Figure 1F). Apparently, increased chromatin association of RECQ1 was also seen following treatment with the DNA inter-strand crosslinking agent mitomycin C (0.5 μg/ml, 24 h) that induces double strand breaks on encounter with progressing replication fork (Figure 1F). When replication fork progression was inhibited by incubating the cells with aphidicolin prior to mitomycin C treatment, chromatin-bound RECQ1 signal was reduced as compared to mitomycin C alone but was greater than the untreated cells (Figure 1F). Thus, stalled and collapsed replication forks induce re-localization of endogenous RECQ1 to the chromatin.

### Aphidicolin treatment induces RECQ1 enrichment at CFS

**Figure 2:**
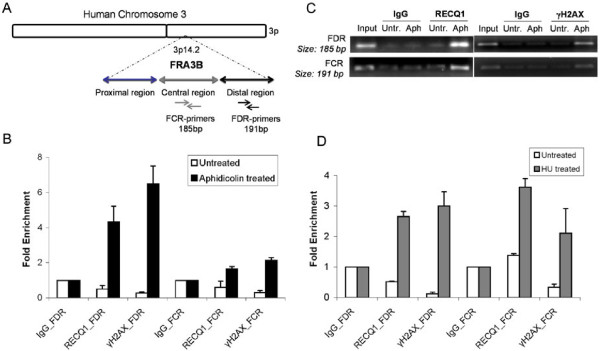
**RECQ1 is recruited to common fragile site FRA3B after treatment with aphidicolin. A**. Genomic organization of the FRA3B region. Primer sets used for qPCR analyses of distal (FDR) and central (FCR) region within the FRA3B locus are indicated. **B**. Quantification of cross-linked FRA3B chromatin immunoprecipitated from HeLa cells using the indicated antibodies. HeLa cells were either untreated or treated with aphidicolin (0.5 μM) for 24 h and then processed for ChIP using a RECQ1-specific antibody. Phosphorylated H2AX (γH2AX) antibody was used as a positive control for FRA3B enrichment, and rabbit IgG served as negative control in ChIP experiments. qPCR was performed with two different sets of primers specific for the central and distal regions within FRA3B locus. Fold enrichment over IgG was determined and is shown for each primer pair for the ChIP. Results are expressed as means ± SEM for at least three independent experiments. **C**. A representative gel of the amplified DNA immunoprecipitated with indicated antibodies. Input represents 1% of the cross-linked chromatin used for ChIP. **D***.* Binding of RECQ1 to FRA3B after treatment with hydroxyurea. Quantification of cross-linked FRA3B chromatin immunoprecipitated from HeLa cells using the indicated antibodies. HeLa cells were either untreated or treated with hydroxyurea (2 mM) for 24 h and processed for ChIP using a RECQ1-specific antibody. γH2AX antibody was used as a positive control for FRA3B enrichment, and rabbit IgG served as negative control in ChIP experiments. qPCR was performed with two different sets of primers specific for the central and distal regions within FRA3B locus. Fold enrichment over IgG was determined and is shown for each primer pair for the ChIP. Results are expressed as means ± SEM for at least three independent experiments. APH, aphidicolin; HU, hydroxyurea.

Replication stress particularly affects genomic loci where progression of replication forks is slow or problematic [2]. To test a putative role of RECQ1 in promoting fork recovery or repair, we examined whether RECQ1 is recruited to CFS since aphidicolin treatment introduces stalled replication forks at fragile sites [5]. In order to determine whether RECQ1 is recruited to stalled replication forks induced at the FHIT region in the aphidicolin-sensitive fragile site FRA3B, HeLa cells were either untreated or treated with 0.5 μM aphidicolin for 24 h. The cross-linked chromatin prepared from each condition was then processed for ChIP by using either control IgG or a specific RECQ1 antibody. Immunoprecipitation using a specific antibody against phosphorylated H2AX (γH2AX) which is known to bind across the FHIT region of FRA3B served as positive control [18,49]. To determine whether RECQ1 occupies FRA3B locus, primers specific for two separate regions in FRA3B fragile locus including the distal aphidicolin induced breakpoints cluster (FDR) located within intron 4 of the *FHIT* gene were used (Figure 2A) [49,50]. As shown in Figure 2B, RECQ1 or γH2AX did not bind the FRA3B locus in untreated cells but were recruited to this fragile site when cells were exposed to aphidicolin. Treatment with aphidicolin induced enrichment of RECQ1 and γH2AX at FRA3B; approximately 4.5- and 6.5-fold enrichment of FDR-specific DNA was obtained in RECQ1 and γH2AX immunoprecipitate as compared to IgG, respectively (Figure 2B, C). RECQ1 and γH2AX immunoprecipitate also contained a relatively modest, but reproducible, enrichment of FCR-specific DNA over IgG, respectively (Figure 2B, C). Thus, aphidicolin treatment induced an increase in RECQ1 occupancy at the FRA3B fragile site in HeLa cells. ChIP experiments using hyroxyurea treated cells (2 mM, 24 h) revealed nearly 2.7- and 3.6-fold enrichment of FDR and FCR-specific DNA in RECQ1 immunoprecipitate as compared to IgG whereas γH2AX immunoprecipitate displayed 3- and 2-fold enrichment of FDR and FCR-specific DNA, respectively (Figure 2D). The relatively reduced binding observed here is likely due to the fact that hydroxyurea and other inhibitors are less specific than aphidicolin in inducing lesions primarily at fragile sites [5].

**Figure 3:**
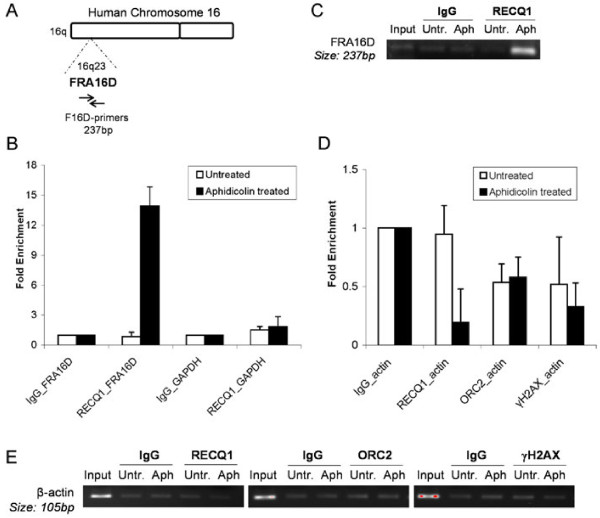
**RECQ1 preferentially binds to FRA16D after treatment with aphidicolin. A**. Genomic organization of the FRA16D region. Primer set used for qPCR analyses of the FRA16D locus is indicated. **B**. Quantification of cross-linked FRA16D chromatin immunoprecipitated from HeLa cells untreated or treated with aphidicolin (0.5 μM) for 24 h using a specific RECQ1 antibody or rabbit IgG. Fold enrichment of FRA16D containing sequences in RECQ1 ChIP was determined by normalizing enrichment obtained with IgG and is shown for both untreated and APH-treated cells. Relative occupancy of RECQ1 at FRA16D versus a non-fragile negative control site GAPDH shows preferential recruitment of RECQ1 to fragile site locus in aphidicolin treated cells. Results are expressed as means ± SEM for at least three independent experiments. **C**. A representative gel of the amplified DNA immunoprecipitated with indicated antibodies. Input represents 1% of the cross-linked chromatin used for ChIP. **D**. qPCR analyses of RECQ1, ORC2 or γH2AX -binding in ChIP experiments to DNA sequence containing β-actin in HeLa cells. **E**. A representative gel of the amplified β-actin sequence immunoprecipitated with indicated antibodies. Input represents 1% of the cross-linked chromatin used for ChIP. APH, aphidicolin.

To ascertain preferential binding of RECQ1 to CFS, we further analyzed RECQ1 immunoprecipitates by qPCR for the enrichment of DNA corresponding to FRA16D, the second most active and aphidicolin-sensitive fragile site in the human genome (Figure 3A) [51]. As control, we also examined two non-CFS DNA sequences located within the GAPDH and β-actin, respectively (Figure 3B, D) [18]. ChIP from untreated HeLa cells did not show enrichment of FRA16D-specific DNA in RECQ1 immunoprecipitate relative to IgG suggesting that RECQ1 does not occupy FRA16D fragile locus in unstressed replicating cells (Figure 3B). Treatment of HeLa cells with aphidicolin induced recruitment of RECQ1 at the FRA16D locus; approximately 14-fold enrichment of FRA16D-specific DNA was obtained in RECQ1 immunoprecipitate as compared to IgG (Figure 3B). In contrast, only 1.5- and 1.8-fold enrichment of control sequence spanning GAPDH was obtained in RECQ1 immunoprecipitate relative to IgG (Figure 3B, C). As compared to IgG, ChIP of RECQ1, γH2AX, or ORC2 did not show enrichment of control sequence spanning β-actin in untreated or aphidicolin treated cells indicating the specificity of respective antibodies used in these experiments (Figure 3D, E).

Collectively, these experiments demonstrate preferential and specific binding of RECQ1 to the two well characterized aphidicolin-sensitive fragile sites FRA3B and FRA16D in response to replication stress.

### RECQ1-depleted cells show aphidicolin sensitivity, defective response to replication stress, and chromosomal fragility

**Figure 4:**
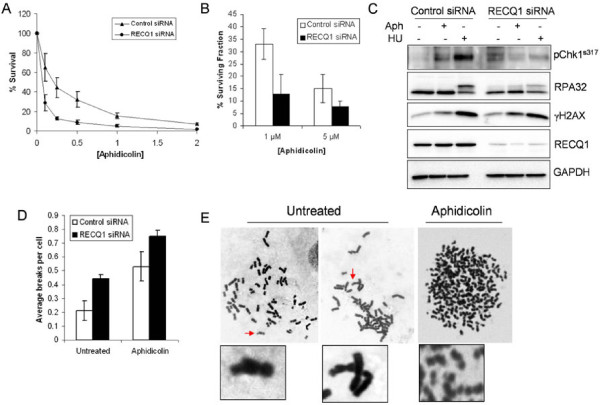
**RECQ1-depletion leads to aphidicolin sensitivity, aberrant replication stress response and chromosomal fragility in HeLa cells. A**. Cells transfected with control or RECQ1 siRNA were exposed in quadruplicate to increasing doses of aphidicolin (μM) or grown in regular complete medium and their survival was measured 72 h later by MTS assay. Percentage of control growth was plotted for each data point, representing the mean ± SD of three independent experiments. **B**. Surviving fraction was determined for control or RECQ1 siRNA transfected cells growing in regular medium or medium supplemented with aphidicolin (μM) by cell count after 72 h and presented as the mean ± SD of three independent experiments. **C**. Detection of the activated forms of Chk1 kinase and the phosphorylated forms of RPA32 and H2AX in untreated or aphidicolin (0.5 μM) treated HeLa cells transfected with control or RECQ1 siRNA. Depletion of RECQ1 by siRNA is also shown. GAPDH serves as loading control. **D***and***E**. Chromosomal breaks/gaps in RECQ1-depleted cells. Metaphase spreads of control or RECQ1 siRNA transected cells (72 h after siRNA) grown in the absence or presence of aphidicolin (0.5 μM, 24 h) were scored for chromosome gaps/breaks. Ten metaphases of each cell condition were analyzed. In untreated condition, RECQ1-depleted cells showed significantly more breaks than control cells (p < 0.05); aphidicolin treatment induced chromosome breaks and gaps in control or RECQ1 siRNA transfected cells (**D**). Representative partial metaphase spreads for RECQ1-depletion is shown (**E**); bottom panel shows an enlarged section. APH, aphidicolin; HU, hydroxyurea.

We previously reported that siRNA mediated depletion of RECQ1 renders HeLa cells more sensitive to camptothecin, an anti-tumor drug that inhibits the topoisomerase-induced DNA breakage-reunion reaction resulting in DNA double strand breaks at stalled replication forks [37]. Given the enrichment of RECQ1 at aphidicolin-sensitive fragile sites, we postulated that RECQ1 may be important in the cellular resistance to aphidicolin. Control or RECQ1 siRNA-transfected cells were exposed to increasing concentrations of aphidicolin and their survival was measured 72 h later by MTS assay. In multiple experiments, RECQ1-depleted HeLa cells displayed increased and dose-dependent sensitivity to aphidicolin when compared to control siRNA-transfected cells (Figure 4A). Increased aphidicolin sensitivity of RECQ1-depleted cells was also observed when proliferation was assayed by cell counting (Figure 4B). This is consistent with the reported sensitivity of RECQ1-depleted cells to HU, and other replication blocking agents [35].

Increased sensitivity to genotoxic agents may be caused not only by defects in DNA repair but also by disruption of cell cycle checkpoint responses to DNA damage [52]. ATR-dependent Chk1 phosphorylation is induced to stabilize replication complexes on stalled forks in cells treated with aphidicolin or hydroxyurea and the RPA coated single-stranded DNA is considered to be the predominant signal for activation of the ATR-Chk1 signal transduction pathway [53]. We examined by Western blotting Chk1 phosphorylation at Ser317 that is central to the normal DNA damage response to replication stress [54]. As compared to control siRNA transfected cells, RECQ1-depleted HeLa cells exhibit constitutively activated Chk1 as shown by phosphorylation at Ser317 in the absence of exogenously induced stress (Figure 4C). Appearance of RPA32 phosphorylation in undamaged RECQ1-depleted cells suggests that the checkpoint response is triggered by DNA structures generated during replication such as collapsed replication forks, long stretches of single-stranded DNA, or unresolved complex intermediates in RECQ1-deficiency. Pharmacological induction of replication stress revealed aberrant checkpoint activation in RECQ1-depleted cells (Figure 4C). Hydroxyurea treatment (2 mM, 24 h) resulted in the phosphorylation of Chk1 at Ser317 and RPA32 in control siRNA transfected HeLa cells (Figure 4C). In contrast, hydroxyurea treatment of RECQ1-depleted cells displayed consistently attenuated phosphorylation of Chk1 at Ser317 and reduced RPA32 phosphorylation (Figure 4C). Reduced Chk1 phosphorylation was also observed in response to aphidicolin treatment (0.5 μM, 24 h) in RECQ1-depleted cells as compared to control HeLa cells (Figure 4C). RPA32 phosphorylation was not evident in control HeLa cells upon arresting replication with aphidicolin [55] and a minimal increase in the signal for RPA phosphorylation was seen in RECQ1-depleted cells as compared to their untreated condition (Figure 4C). We observed a modest increase in signal for γH2AX in untreated and aphidicolin treated RECQ1-depleted cells as compared to control siRNA transfected cells (Figure 4C).

Partial inhibition of DNA synthesis by aphidicolin results in incomplete replication in late replicating chromosomal areas such as fragile sites leading to visible breaks in metaphase chromosomes [56]. We therefore examined whether RECQ1-depletion increases the basal and aphidicolin-induced metaphase chromosome breaks. Depletion of RECQ1 led to a significant increase in the number of chromosome breaks per cell even in the absence of aphidicolin when compared to the control siRNA (Figure 4D). This is consistent with spontaneous chromosome breaks and gaps observed in RECQ1 knockout mouse embryonic fibroblasts [36] and a recent report using stable knockdown of RECQ1 in human cells [35].

Given the demonstrated ability of RECQ1 to act upon replication fork like structures in vitro [33,35], these results indicate that depletion of RECQ1 as such may contribute to replication fork collapse and also diminish recovery from replication arrest.

## Discussion

Here we show that conditions of replication stress, specifically aphidicolin-induced DNA polymerase α, δ and inhibition, and hydroxyurea-mediated inhibition of ribonucleotide reductase, induce a preferential accumulation of RECQ1 at the lamin B2 origin in HeLa cells. Consistent with a role in promoting fork recovery or repair, we find that RECQ1 is enriched at two major fragile sites, FRA3B and FRA16D, where replication forks have stalled following aphidicolin treatment. Moreover, RECQ1-depletion results in diminished checkpoint activation in response to replication stress, increased sensitivity to aphidicolin and chromosomal instability. These results suggest that RECQ1 is important for maintaining genomic integrity when DNA replication forks are slowed by hydroxyurea or aphidicolin and promote efficient recovery from replication stress.

Results from Thangavel et al proposed that RECQ1 is assembled at origins at the start of bidirectional replication and is subsequently lost from the origin perhaps still tracking along with the newly formed replisome following origin firing [34]. Consistent with this, we found a significant enrichment of RECQ1 at origins when replication fork progression was inhibited by treatment with hydroxyurea or aphidicolin. Furthermore, our ChIP data show differential recruitment of RECQ1 at the lamin B2 origin in response to treatment with aphidicolin (about 30-fold) or hydroxyurea (about 11-fold). Aphidicolin has been shown to have little effect on the activation or initiation of replication origins and induces uncoupling of replication machinery [57]; it is likely that RECQ1 recognizes the long stretch of single-stranded DNA produced due to functional uncoupling of the replicative polymerase and helicase complexes following aphidicolin treatment. Indeed, we found that endogenous RECQ1 displays preferential binding to CFS as compared to non-fragile control DNA especially after cells are treated with aphidicolin. CFS represent single-stranded unreplicated chromosomal regions caused by stalled or collapsed replication forks [5]. This notion has been substantiated by investigation of replication timing [58] and the evidence of involvement of checkpoint proteins ATR [59,60], BRCA1 [61], SMC1 [62] and FANCD2 [63] in fragile site stability. Additionally, CFS sequences including FRA16D are characterized by high AT content, AT-rich mini satellite repeats and their tendency to form secondary structures [64]. Human RecQ proteins have demonstrated ability to resolve a variety of non-B DNA secondary structures [65]. It is yet unknown whether RECQ1, like WRN [16], can resolve the predicted cruciform structures at FRA16D that stall replication fork progression and contribute to chromosome breakage; however, the fact that RECQ1 was also enriched at FRA3B that is devoid of mini- or microsatellite [6] indicates that the secondary structures alone may not be a sufficient structural element for RECQ1 binding to the fragile sites. WRN functions at fragile sites are critical but it has not yet been shown whether WRN is recruited to the fragile site loci in vivo, and whether it can distinguish fragile and non-fragile regions under replication stress. RecQ proteins are known to form multi-protein complexes to execute their functions [14,66], and it is conceivable that yet unknown protein partners of RECQ1 recruit and/or mediate its functions at stalled and broken forks at fragile site loci and elsewhere in the genome.

Stalled replication fork activates checkpoint signaling pathways to coordinate cell cycle progression with repair of damage, ensuring the integrity of the genome [52,67]. ATR and Chk1-dependent checkpoints prevent excessive formation of DNA double strand breaks during replication arrest [54]. RPA protein complex, consisting of three subunits RPA72, RPA32 and RPA14, is a first sensor of replication-associated damage and is thought to signal activation of ATR and thereby trigger an intra-S checkpoint [68]. Depletion of RECQ1 led to spontaneous phosphorylation of RPA32 and activation of Chk1; however, RECQ1-depleted cells are defective in triggering replication stress response and exhibit sensitivity to replication blocking agents [35]. During normal DNA replication, optimal binding of RECQ1 to the origins may ensure appropriate and accurate genome duplication during S-phase. Loss of RECQ1 leads to aberrant elongation of progressing replication forks [34] which may lead to activate the checkpoint response. The fork promoting activity of RECQ1 could be especially important at naturally occurring DNA sequences such as fragile sites that are at increased risk for stalling the replication fork even in the absence of external replication stress. Our data implicate that RECQ1 also participates in relaying signals of fork stalling that help coordinate a faithful cell cycle and recovery from replication stress. RECQ1 may contribute to mutational avoidance in unperturbed and deliberately stalled replication.

Chromosome breaks observed upon acute depletion of RECQ1 is consistent with a recent finding that depletion of RECQ1 activates DNA damage signaling cascade and accumulates replication-induced double strand breaks [35]. Conditions that slow replication along the entire genome, such as aphidicolin treatment, lead to double strand break formation as a result of fork stalling and collapse at fragile sites and activate the double strand break repair pathways [69]. Homologous recombination is a major mechanism utilized to repair stalled or collapsed replication forks [70]. Importantly, homologous recombination mechanisms triggered by replication arrest differ from those involved in repairing classical two ended DNA double strand breaks [71]. Thus, although the repair of I-Sce induced double strand break was not significantly modulated by RECQ1-deficiency [47], a role for RECQ1 in recombination repair of replication-induced double strand breaks remains possible. In vitro activity of RECQ1 to unwind synthetic replication fork and catalyze strand exchange indicates its potential ability to form and subsequently branch migrate Holliday junction like recombination structures generated during template switching at the stalled forks [35,71]. It is conceivable that in the absence of RECQ1, recombinogenic DNA structures at arrested forks are repaired via homologous recombination.

Fragile loci often coincide with chromosomal breakpoints in tumors [5,72]. Given the elevated proliferation status of tumor cells, proteins involved in the cellular response to replication stress are likely to act as caretakers of the genome during tumor development [12]. A mutation in RECQ1 has not been linked to a human disease yet, but an Oncomine database search shows that RECQ1 is over-expressed in many clinical cancer samples compared to matched normal samples (Additional file 1: Figure S1). Similar trend of RECQ1 over-expression across various tumors is presented by another web database search (http://medicalgenome.kribb.re.kr/GENT/). It is plausible that cancer cells position RECQ1 on to specific genomic loci so as to cope with increased replication-mediated DNA damage during rapid cell division; in normal cells, RECQ1 can act as a tumor suppressor by facilitating DNA repair and preventing mutations. This notion is consistent with the observation that RECQ1 is uniquely important for the proliferation of cancer cells [37,40,41]. DNA breakage within CFS is thought to be a consequence of failing to complete replication and/or resolving the arrested forks prior to the end of telophase and chromosome segregation [19]. Recent reports have also suggested that chromosomal breaks occur at fragile sites because these loci are late replicating and origin poor [73-75]. Whether chromosomal breaks in RECQ1-deficient cells occur at CFS remains to be examined, but recruitment of RECQ1 at FRA3B and FRA16D suggests that RECQ1 either prevents replication fork stalling within origin poor regions or resolves replication problems at these CFS. Future experiments will examine what functional sub-modules of DNA replication are associated with RECQ1 at specific genomic loci and how it participates in dynamic response to challenges during DNA replication. Present findings together with the in vitro results indicate that impaired response to replication stress contributes to genomic instability in RECQ1-deficient cells.

## Conclusions

RECQ1, to our knowledge, is the first human RecQ helicase shown to bind CFS. Overall, our results provide new insight to RECQ1 functions and contribute to dissecting unique and overlapping roles of human RecQ homologs in facilitating replication fork progression through natural impediments in genome.

*Note added in proof:* While this work was under review, a study by Berti et al. also reported a role of RECQ1 in replication fork restart [76].

## Materials and methods

### Cell culture, drug treatment and siRNA transfection

Human HeLa (ATCC) were grown in Dulbecco's modified Eagle's medium (DMEM) (Invitrogen) supplemented with 10% fetal bovine serum (Hyclone Laboratories), 100 U/ml penicillin and 100 μg/ml streptomycin (Invitrogen). Cells were grown in a humidified 5% CO_2_ incubator at 37°C. To induce replication stress, exponentially growing cells were treated with hydroxyurea (Sigma), aphidicolin (Calbiochem), mitomycin C (Sigma) as indicated. Depletion of RECQ1 was achieved by transfecting HeLa cells with a scrambled control siRNA or a SMARTpool of four distinct siRNA species targeting different regions of RECQ1 mRNA (siGenome SMARTpool, Dharmacon) at a final concentration of 10 nM using Lipofectamine 2000 transfection reagent as per the manufacturer’s instructions (Invitrogen). Knockdown specificity and efficacy of RECQ1 smartpool siRNA was evaluated by real-time PCR and Western blotting.

### Chromatin immunoprecipitation (ChIP)

HeLa cells were cultured overnight at a density of 1 × 10^7^ per 15 cm diameter dish and subjected to either no treatment or treatment with 0.5 μM aphidicolin for 24 h. Chromatin and proteins were cross-linked by incubating cells in 1% formaldehyde for 15 min at room temperature and the reaction was stopped by 10 min incubation with 125 mM glycine. Cells were collected and washed sequentially with solution I (10 mM HEPES [pH 7.5], 10 mM EDTA, 0.5 mM EGTA, 0.75% Triton X-100) and solution II (10 mM HEPES [pH 7.5], 200 mM NaCl, 1 mM EDTA, 0.5 mM EGTA). The cell pellets were resuspended in 2ml lysis buffer (25 mM Tris-HCl [pH 7.5], 150 mM NaCl, 0.1% SDS, 1% Triton X-100, 0.5% deoxycholate freshly supplemented with 1X protease inhibitor cocktail (Roche)) and sonicated on ice by 10 s pulses at 30% of maximal power on a Misonix 2000 sonicator (Misonix). This sonication method consistently yielded chromatin fragments corresponding to an average DNA length of 400-1000 bp as checked on a 1.5% agarose gel. After centrifugation at 20,000X g for 15 min to remove any debris, the supernatant was pre-cleared with protein-G-sepharose/salmon sperm DNA beads (Millipore) at 4°C for 1 h. For each immunoprecipitation, 600 μl (equivalent of 3 × 10^6^ cells) of the pre-cleared chromatin was incubated overnight at 4°C with 3 μg of antibodies specific for either RECQ1 (Bethyl Lab, A300-450A), γH2AX (Millipore, 05-636, clone JBW301), or ORC2 (Enzo Life Science, ADI-KAM-cc235); antibodies were confirmed for their immunoprecipitation specificity using Western blot. A reaction containing an equivalent amount of rabbit IgG was included as the background control. 10% of the pre-cleared chromatin was set aside as input control. Antibody-chromatin complexes were pulled down by adding 50 μl of protein-G-sepharose/salmon sperm DNA beads and incubated for 2 h at 4°C. The beads were washed for 10 min each with the following solutions: lysis buffer (as mentioned above), high-salt wash buffer (0.1% SDS, 1% Triton X-100, 2 mM EDTA, 20 mM Tris-HCl [pH 8.1], 500 mM NaCl), LiCl wash buffer (250 mM LiCl, 1% NP-40, 1% deoxycholate, 1 mM EDTA, 10 mM Tris-HCl [pH 8.0]), and TE buffer (10 mM Tris-HCl [pH 8.0], 1 mM EDTA). Finally, DNA was eluted with elution buffer (1% SDS, 100 mM NaHCO_3_). Eluates were incubated at 65°C for overnight with the addition of 5 M NaCl to a final concentration of 200 mm to reverse the formaldehyde cross-linking and digested at 55°C for 3 h with proteinase K at a final concentration of 50 μg per ml. Following phenol/chloroform extraction and ethanol precipitation, sheared DNA fragments served as template in qPCR analysis. qPCR were performed using Taq Universal SYBR Green Supermix (Bio-Rad) with technical triplicates, and threshold cycle numbers (C_t_) were determined with an iQ5 thermal cycler (Bio-Rad). Fold enrichment of the targeted genomic sequences were calculated over IgG as: fold enrichment = 2^-(Ct^_IP_^− Ct^_IgG_^)^, where Ct_IP_ and Ct_IgG_ are mean threshold cycles of PCR done in triplicates on DNA samples immunoprecipitated with specific antibody and control IgG, respectively. All qPCR reactions were also checked by melt curve analyses and agarose gel electrophoresis to confirm the presence of a single specific product. The sequences of the qPCR primers are listed in Table 1.

**Table 1:** Primers used for quantitative real-time PCR analyses

**Name**	**Sequence**	
B13	Forward	5′- CCCCAGGGAGTAGGTTGTGA-3′
	Reverse	5′- TGTTATTTGAGAAAAGCCCAA-3′
B48	Forward	5′- CTCCACCCCCAAGGAAAAAG-3′
	Reverse	5′-GGCAGGGTCCCATGCA-3′
FCR	Forward	5′-TGTTGGAATGTTAACTCTATCCCAT-3′
	Reverse	5′-ATATCTCATCAAGACCGCTGCA-3′
FDR	Forward	5′-CAATGGCTTAAGCAGACATGGT-3′
	Reverse	5′-AGTGAATGGCATGGCTGGAATG-3′
FRA16D	Forward	5′-TCCTGTGGAAGGGATATTTA-3′
	Reverse	5′-CCCCTCATATTCTGCTTCTA-3′
GAPDH	Forward	5′-CCCTCTGGTGGTGGCCCCTT-3′
	Reverse	5′-GGCGCCCAGACACCCAATCC-3′
β-actin	Forward	5′-GACGCAGGATGGCATGGG-3′
	Reverse	5′-ACGCCTCTGGCCGTACCAC-3′

### Flow cytometry

Cells were collected by trypsinization and fixed with 70% ethanol for 30 min. Cells were subsequently washed in PBS, treated with RNase in PBS, and resuspended in PBS with 4 μg/ml propidium iodide. Cells were analyzed on a FACSCalibur™ flow cytometer from BD Science using ModFit LT software.

### Biochemical fractionation and Western blotting

Chromatin enriched fractions were prepared as previously described [47] from cells that were either untreated or treated with indicated concentration of hydroxyurea or aphidicolin. To obtain total extracts, cells were lysed in RIPA buffer (50 mM Tris-HCl [pH 8.0], 150 mM NaCl, 1% NP-40, 0.5% sodium deoxycholate, 0.1% SDS) containing cocktail of protease and phosphatase inhibitors (Roche). Equal amounts of total protein for each sample was run on 8-16% SDS-PAGE and transferred to PVDF membrane for immunoblotting with antibodies for phospho-Chk1 Ser 317 (Cell Signaling, 2344), γH2AX (Cell Signaling, 2577), RPA32 (Bethyl Lab, A300-244A), RECQ1 (Bethyl Lab, A300-450A), GAPDH (Cell Signaling, 2118), and Histone H3 (Cell Signaling, 4499); all antibodies were used at 1:1000 dilution.

### Cell proliferation assay

Cells (24 h after siRNA transfection) were seeded in quadruplicate at a density of 3000 cells/well in 96-well plates and allowed to adhere for 16 h and subsequently exposed to increasing concentration of aphidicolin (0-5 μM) and allowed to grow at 37°C for 3 days in 5% CO_2_. Cell proliferation was determined by CellTiter 96® AQueous Non-Radioactive Cell Proliferation Assay (MTS) (Promega). Additionally, cells were transfected with the indicated siRNA, split 1:4 onto a multiwell plate, and grown in the presence or absence of aphidicolin for further 72 h. The cells in one well were counted every 24 h for the duration of the experiment.

### Metaphase chromosome analyses

Cells, 48 h after transfection, were grown in the presence or absence of aphidicolin (0.5 μM) for 24 h and treated with 0.5 μg/ml colchicine for 4 h at 37°C before collection. To prepare metaphase spreads, cells were resuspended in hypotonic solution (0.06 M KCl) for 15 min at room temperature, and then fixed with 3:1 (vol/vol) methanol-glacial acetic acid. Fixed cell suspension was dropped onto precleaned microscope slides and air-dried overnight. Metaphase chromosomes were visualized by Giemsa staining. Images were documented with a Nikon microscope and at least 10 metaphase chromosome spreads per treatment were scored in a blinded fashion.

## Competing interests

The authors declare that they have no competing interests.

## Authors’ contributions

XL carried out the chromatin interaction studies, participated in the chromosome analyses, analyzed the data and drafted the manuscript in parts. SP carried out the knockdown experiments and Western blotting. TH performed and AL supervised FACS analyses. SS conceived of the study, participated in its design and coordination, and helped to analyze the data and draft the manuscript. All authors read and approved the final manuscript.

## Supplementary Material

Additional file 1: Figure S1 RECQ1 gene expression across many tumor-normal datasets. The Oncomine™ (Compendia Bioscience, Ann Arbor, MI) database (http://www.oncomine.org/) was used to determine how many datasets indicate up-regulation of RECQ1 (also known as RECQL) in cancer versus normal. Top 10% of genes in the given dataset were considered differentially expressed and the number of datasets pointing to up or down-regulation of RECQ1 was counted. A total of 42 out of 391 differential expression analyses included RECQ1 in the top 10% up-regulation list while only 2 did in the top 10% down-regulation list. Click here for file
