# Supplementary material for: Replication stress induces specific enrichment of RECQ1 at common fragile sites FRA3B and FRA16D
Source: Mol Cancer. 2013 Apr 22;12:29. doi: 10.1186/1476-4598-12-29 (PMC3663727; doi:10.1186/1476-4598-12-29)
Supplement: Additional file 1: Figure S1 — RECQ1 gene expression across many tumor-normal datasets. The Oncomine™ (Compendia Bioscience, Ann Arbor, MI) database (http://www.oncomine.org/) was used to determine how many datasets indicate up-regulation of RECQ1 (also known as RECQL) in cancer versus normal. Top 10% of genes in the given dataset were considered differentially expressed and the number of datasets pointing to up or down-regulation of RECQ1 was counted. A total of 42 out of 391 differential expression analyses included RECQ1 in the top 10% up-regulation list while only 2 did in the top 10% down-regulation list. [file 1476-4598-12-29-S1.pdf]

Supplementary Figure Legend

Supplementary Figure 1. RECQ1 gene expression across many tumor-normal datasets. The Oncomine™ (Compendia Bioscience, Ann Arbor, MI) database (http://www.oncomine.org/) was used to determine how many datasets indicate up-regulation of RECQ1 (also known as RECQL) in cancer versus normal. Top 10% of genes in the given dataset were considered differentially expressed and the number of datasets pointing to up or down-regulation of RECQ1 was counted. A total of 42 out of 391 differential expression analyses included RECQ1 in the top 10% up-regulation list while only 2 did in the top 10% down-regulation list.

Disease Summary for RECQL

Analysis Type by Cancer	Cancer vs. Normal	
Bladder Cancer		
Brain and CNS Cancer	6	
Breast Cancer	1	
Cervical Cancer	1	
Colorectal Cancer	5	
Esophageal Cancer		
Gastric Cancer		
Head and Neck Cancer	4	
Kidney Cancer	6	
Leukemia		
Liver Cancer		
Lung Cancer		1
Lymphoma	4	1
Melanoma		
Myeloma		
Other Cancer	6	
Ovarian Cancer	1	
Pancreatic Cancer	1	
Prostate Cancer		
Sarcoma	8	
Significant Unique Analyses	42	2
Total Unique Analyses	391	

1 5 10 10 5 1

%

Cell color is determined by the best gene rank percentile for the analyses within the cell.
